# Editorial

**Published:** 1869-09

**Authors:** 


### Editorial.

MERCY HOJPITAL.—The account of the new Mercy Hospital building, and the address at the laying of the corner stone, given in another part of this number of THE EXAMINER, are copied from the *Daily Republican*, of this city. The building is rapidly progressing, the walls being nearly ready for the roof. Most of our readers will be pleased to learn that arrangements have been made by which a new building for the Chicago Medical College will be erected on a part of the Hospital grounds, early next year.

CHICAGO HOSPITAL FOR WOMEN AND CHILDREN.—This institution has been removed to 402 North State street. We understand this has been selected as its permanent location. The Woman's Hospital has become one of the established institutions of this city. It has a rather formidable array of *consulting* physicians, surgeons, obstetrians, and opthalmologists, but is practically under the immediate care of Miss Doctoress THOMPSON.

MEDICAL COLLEGES.—We have received the Announcements of most of the Medical Colleges of the country in reference to the annual coursesof instruction to be commenced in October and November next. Very few of them indicate any change from the usual routine of short lecture terms and heterogenous crowding of instruction on all the principal branches of medicine upon the same students, in the compass of eighteen weeks. It might be well expressed in "*large promises to be fulfilled in a remarkably short period of time, with unequalled facilities for accomplishing the work.*" Indications of progress are, however, visible in some quarters. A new school has been organized in St. Louis, called the "Saint Louis College of Physicians and Surgeons," with a full faculty, five months lecture term and a division of the branches into junior and senior courses, with an optional third course. This is a full recognition of the principle of consecutive order of studies and division of the classes, and is consequently a long step in the right direction. We hope the faculty will be sustained by the profession, because, if they are, they will soon follow the example of the Chicago Medical College, and adopt the complete system of *three consecutive courses of six months each*, with a fair standard of preliminary education, which is what the profession demands, and nothing short of which can afford to the student the facilities he needs for gaining an adequate knowledge of medicine. It is the plain duty of the profession everywhere to promptly sustain those colleges that do break over the old and absurd routine of short terms and heterogenous and repetitional teaching, simply because such a course is the only one that will speedily ensure the universal adoption of theimprovements in our system of medical college instruction, which all acknowledge are so much needed.

---

NATIONAL LIBRARY.—We call the special attention of our readers to the following circular, and hope each one will contribute something to so desirable an object.—[EDITOR.]

WASHINGTON, D. C.

The Medical Profession, and scholars generally, are aware of the ephemeral form in which most of the early American contributions to the literature of medicine were given to the world, and, indeed, in which many of the more recent are being published. This condition of much of our professional literature is deeply regretted by all, and particularly by those whose taste and research lead them to refer to this class of works, when the fact is made apparent that whole editions of tracts and books have entirely perished through neglect. With a view to provide against such a contingency, and preserve, for the benefit of the profession, in some accessible and central locality, copies of all home medical publications, the American Medical Association, at its annual meeting in May last, resolved to establish at Washington, D. C., a Library or Repository of American medical works, to which it is believed all the current medical literature of our country will be cheerfully, promptly, and constantly contributed.

It is designed that this repository shall contain copies of every contribution by American physicians to the literature and science of medicine, from the earliest settlement of our country, no matter how or where published, including all the books, pamphlets, journals, and even unpublished manuscripts that can be collected.

Nearly all physicians have some book or pamphlet of the character indicated, which may contain facts relative to the diseases of his section, published nowhere else, which they can contribute without inconvenience, and which of itself is of trifling value, yet when many such treatises are assembled together from all parts of our country, embracing its nosology from the earliest period of its settlement, they will form a collection of the greatest importance to the profession.

The Librarian of Congress has kindly consented to receive and preserve as a special deposit, in the Government fire-proof building, any collection of medical works the American Medical Association may make, and will catalogue and keep them in condition to be readily consulted. The accommodation thus offered the Association for accumulating and preserving its library free of cost is generous and most encouraging. Gentlemen having scarce and valuable American medical publications, will not hesitate to deposit them in such a safe, central, and national repository, where they will be preserved from destruction and their usefulness secured to the profession.

An appeal for contributions to this library is now made, personally and distinctly, to each and every American Physician, Medical Publisher, and Editor, to deposit copies of their works in this repository, where they will be carefully kept for reference and catalogued with the name of the donor.

We, the undersigned, members of the American Medical Association, having been selected to carry into effect, as far as practicable, the resolution of the Association to establish a Library, have now completed all the necessary arrangements for the reception and preservation of those books which may be sent to our care. Contributions of the class of works mentioned are therefore respectfully and earnestly solicited from every source. Packages may be sent by mail or by Adams' express to either of us, which will be promptly acknowledged on reception, and a record of titles kept. The library mark of the Association will be pasted on the inside of the cover of each volume, which will contain also the name of the donor.

Hoping that you may further the project to the extent of adding at least your own productions,

We remain, respectfully,

ROBERT REYBURN, M.D., *Librarian.*
JOSEPH M. TONER, M.D., *Library Committee.*

---

ETHERIZATION AND BOSTON.—That Bostonians are remarkably sensitive on the subject of Etherization and its dangers, is evident from the number of certificates and letters we publish in the present number of THE EXAMINER, concerning the statement made by Dr. POWELL, at the recent meeting of the Illinois State Medical Society.

If their sensitiveness had not been remarkable, they would probably have delayed the certificate business long enough to have, at least, asked for a note of explanation either from the Secretary of the Society or Dr. POWELL, or both. The history of the matter, so far as relates to the proceedings of the Illinois State Medical Society, is as follows: The comparative danger of different anæsthetics being the subject immediately under discussion, Dr. POWELL related the fact, that while on a visit to one of the hospitals in Boston, he saw ether administered to a patient who was to undergo some operation on the urethra. He said the patient passed under the influence of the anæsthetic, and the surgeon commenced the operation, when it was soon noticed that respiration had ceased. Immediate efforts were made to resuscitate the patient, and with partial success, but not so complete but that he was soon wrapped up and removed from the room. Dr. POWELL called at the hospital on the following day, and on inquiring of the Assistant-Surgeon, understood him to say that the patient died on the previous night. Dr. POWELL simply stated what he supposed to be the facts, without saying whether the anæsthetic killed the patient or not. The Secretary, in his minutes, put down the inference that would be drawn from Dr. POWELL'S statement, instead of the statement itself. The only important error of fact in the statement of Dr. POWELL, and on which all errors of inference depended, was the misunderstanding or *misinformation* in regard to the patient having died the evening after the operation. It appears from the statement of Dr. CHEEVER and the hospital records, that he lived a considerable time after the operation, and died with disease of the heart. Would it not be well for all surgeons to ascertain whether patients have organic disease of the heart, before they give the anæsthetic, and when they operate publicly, to inform such practitioners and siudents as may be looking on, when such complication exists? Would not such precaution both lessen the number of accidents to patients and the liability to false inferences by others?

MR. EDITOR,—In the *Chicago Medical Examiner* for July, 1869, p. 407, in a "report of the nineteenth Annual Meeting of the Illinois State Medical Society, commencing May 18, 1869, in the City of Chicago"; on Thursday morning, the third day, in a general discussion on Chloroform and Ether, there occurs the following paragraph:—

"Dr. E. Powell stated the fact that a few years since he saw the Ether administered to a patient in the Hospital at Boston, Massachusetts, who died directly from the effects of the inhalation."

If by the Hospital at Boston, Massachusetts, is meant the Massachusetts General Hospital, the undersigned, comprising all the past and present surgeons of that Institution, now living (with the exception of Dr. H. J. Bigelow, who is temporarily absent from the City), desire to say, that no patient ever died from the inhalation of Ether in that Hospital, nor are they cognizant of any such occurrence in Boston or elsewhere.

S. D. TOWNSEND, *late Surgeon M. G. H.*

HENRY G. CLARK,

SAMUEL CABOT,

GEO. H. GAY,

R. M. HODGES,

ALGERNON COOLIDGE,

} *Attending Surgeons.*

BENJ. S. SHAW, *Resident Physician.*

*Boston, July* 29, 1869.

Mr. Editor,—My attention having been called to a statement made by Dr. E. Powell, at the last annual meeting of the Illinois State Medical Society, and published in the *Chicago Medical Examiner*, Vol. X., No 7, July, 1869, at page 407, to the effect that "he saw, a few years since, ether administered to a patient in the [City] Hospital at Boston, Mass., who died directly from the effects of the inhalation," I beg leave to say: —That I remember perfectly that on one occasion Dr. Powell was present in the amphitheatre of the City Hospital. I was operating on a patient, by Syme's operation, for perineal section. He became very seriously affected under the etherization; the operation was suspended; stimulants and artificial respiration were employed; the patient rallied; the operation was resumed and completed without further etherization, and the patient subsequently lived *twenty days*, and died of pyæmia. He was found to have had a serious valvular disease of the heart. He was an old man, enfeebled by extravasation of urine. For the details I would refer to the Hospital Records, Vol. XV., p. 104.

As the statement of Dr. Powell was wanting in precision and void of fact, I have thought it right to place the whole truth before you. No case has yet occurred at the City Hospital of death from an anæsthetic. Sulphuric ether (Squibb's, or Powers & Weightman's) is the anæsthetic employed in this Hospital.*

Respectfully, D. W. Cheever, M.D.
One of the Surgeons of the Boston City Hospital.
*Boston*, *August*, 1869.

From Vol. XV., Surgical Records of the Boston City Hospital, p. 104 *et seq.*:—

"Feb. 18, 1868. Edward Boice, laborer, æt. 50, No. 1 Friend St. Ten years since, contracted gonorrhœa. Five years ago, had stoppage of urine. Three years ago, had another stoppage, water coming away by drops greater part of the time, causing severe pain. Fourteen days ago, began to be troubled with stoppage and increased pain, which produced great straining; to micturate was almost impossible. Eight days ago, first noticed a small lump just anterior to the scrotum, which enlarged on straining (on under side of penis). Since that time the whole organ has been infiltrated, either by urine or serum, so that it was twice the normal size when seen to-day. About two inches of the base is as hard as a stick, the rest œdematous.

*As we are about going to press, we would here say that we wrote to Dr. Powell, a week ago yesterday, asking him for further advices as to what he saw in the case he alludes to in "the Hospital in Boston, Massachusetts." His reply has not yet come to hand.—Editor, August 10th, 1869.

Phymosis. The scrotum and perinæum are not affected. His urine had entirely stopped, so that he had not urinated for the last twenty-four hours. Bladder very much distended. In great misery.

"Seen by Dr. Cheever at 12, M., and it was found that the urethra was entirely closed about two inches from the meatus. Stricture very firm indeed. He punctured the bladder through the rectum, and drew off about two pints of urine. Removed the canula, and allowed it to dribble through into the rectum. Made three incisions in the base of the penis, and ordered him to be kept under opiates if in pain.

"Feb. 21st.—Etherized and placed in the lithotomy position. A sharp-pointed, grooved staff was passed into the urethra, but it was found impossible to insert it over an inch. An incision was then made down upon the urethra anterior to the scrotum, and the urethra laid open nearly two inches.

This allowed the staff to pass along beneath the scrotum, but another obstruction and a false passage were found. At this time the patient became very feeble. Respiration stopped. Pulse very slow and feeble—at last it stopped. Place him upon his back, and by persevering in artificial respiration, by throwing both arms about the head and then down against the sides, succeeded in restoring patient to life. No more ether was given. An incision was made down upon the bulb, urethra found and opened, and an elastic catheter passed into the bladder and kept there.

"Evening visit. Good pulse, but slightly delirious. Water passes freely through the catheter.

"Feb. 22.—Passed a comfortable night and is rational now. Is comfortable. * *

"Feb. 27.—Rested very well during the night. Swelling of the knee increased. Effusion into the joint. Whole leg nearly to the hip more or less œdematous. Blush of knee and leg increased. Veins of thigh loaded. Complains of pain in the knee. No chills or sweats. Wound in perinenm and penis looks well.

"March 12.—A very restless night. Hiccoughs and considerable pain. Abdomen very tympanitic and a slight blush just above the pubes. Pulse feeble. Appetite poor. Evidently failing. Emesis. Evening—Failing. But very little urine. Pulse feeble. Countenance pale. 10.40 P.M. Dead. Caused probably by pyæmia and peritonitis. No autopsy."

A true copy, GEORGE B. STEVENS, *House Surgeon.*

### Mortality for the Month of July, 1869:—

| | |
|---|---|
| Accident, burned | 2 |
| " brain, concussion of, | 1 |
| " drowned | 13 |
| " fall | 2 |
| " fracture of skull | 1 |
| " by machinery | 2 |
| " dose of aconite | 1 |
| " unloading vessel | 1 |
| " rupture of blood vessel | 1 |
| " railroad | 4 |
| Anæmia | 2 |
| Aneurism adorta, rupture of | 1 |
| Anus, imperforate | 1 |
| Apoplexy | 1 |
| Births, premature | 18 |
| " still | 47 |
| " tedious | 1 |
| Bowels, inflammation, | 6 |
| " obstruction of | 2 |
| Brain, congestion of | 5 |
| " stomach and bowels, inflammation of | 1 |
| " inflammation | 5 |
| " softening of | 1 |
| Bronchitis | 2 |
| " and whooping-cough | 1 |
| Cancer of femoral artery and gangrene | 1 |
| Cancer, ventricle | 1 |
| Childbirth | 2 |
| Chlorosis | 1 |
| Cholera infantum | 186 |
| " " and teething, | 1 |
| Cholera morbus | 4 |
| Convulsions | 72 |
| " and teething, | 1 |
| Croup | 4 |
| " diphtheretic | 1 |
| Consumption | 39 |
| " and gastritis, | 1 |
| Cyanosis | 2 |
| Debility | 8 |
| " general | 3 |
| Delirium tremens | 2 |
| Diarrhœa | 31 |
| " and convulsions, | 4 |
| " and teething | 1 |
| " and whooping-cough | 1 |
| " chronic | 3 |
| Diphtheria | 11 |
| Dropsy | 7 |
| Dyspepsia | 1 |
| Dysentery | 18 |
| " acute | 2 |
| " and bronchitis, | 1 |
| Empyema | 1 |
| Encephalitis | 2 |
| Entero-colitis | 2 |
| Enteritis | 15 |
| Exhaustion | 1 |
| Erysipelas | 2 |
| Fever, congestive | 1 |
| " puerperal | 3 |
| " remittent | 3 |
| " scarlet | 61 |
| " " malignant, | 5 |
| " " and convulsions | 2 |
| " " and abscess neck, | 1 |
| " typhoid | 17 |
| " typhus | 1 |
| Gangrene, traumatic, result of accident | 1 |
| Gastritis | 2 |
| Gastro-enteritis | 1 |
| Heart, disease of | 9 |
| " and liver, disease of | 1 |
| " dropsy of | 1 |
| " hypertrophy of, | 2 |
| " rupture of | 1 |
| " organic disease of | 3 |
| Hemipligia | 1 |
| Hepatitis, chronic | 1 |
| Hydrocephalus | 8 |
| " acute | 3 |
| " and teething, | 1 |
| Inanition | 15 |
| Intemperance | 1 |
| Jaundice | 1 |
| Kidneys, Bright's disease of | 2 |
| Kidneys, inflammation of | 2 |
| Laryngitis | 1 |
| Liver, cirrhosis and enlargement of | 1 |
| Liver, hæmorrhage of, | 1 |
| Lungs, congestion of | 1 |
| Manslaughter | 2 |
| Measles | 34 |
| " and pneumonia | 2 |
| " and whooping-cough | 1 |
| Meningitis | 5 |
| " cerebro-spinal, | 4 |
| " tubercular | 5 |
| Mumps | 1 |
| Nutrition, defective | 2 |
| Old age | 6 |
| Parotitis | 1 |
| Paralysis | 2 |
| " and brain, softening of | 1 |
| Pericarditis | 1 |
| Peritonitis | 6 |
| " puerperal | 3 |
| Pneumonia | 16 |
| " broncho | 1 |
| " and measles, | 1 |
| " and conjestion of lungs | 1 |
| Rheumatism | 1 |
| Scrofula | 1 |
| Small-pox | 2 |
| Spine, disease of | 2 |
| Suicide | 2 |
| Sun-stroke | 3 |
| Syphilis, hereditary | 1 |
| Tabes, mesenterica | 25 |
| Teething | 15 |
| " and convulsions, | 3 |
| " and diarrhœa | 3 |
| Thigh, amputation of, from wound of knee joint | 1 |
| Uremia | 1 |
| Uterus, cancer of | 1 |
| Whooping-cough | 11 |
| " and convulsions, | 2 |
| " and teething | 1 |
| Worms, stomach | 1 |
| Unknown | 2 |
| Total | 815 |

COMPARISON.

Deaths in July, 1869, 815 | Deaths in June, 1868, 892 | Decrease, 82
Deaths in June, 1869, 434 | Increase, 381

AGES.

| | | | | | |
|---|---|---|---|---|---|
| Under 1 | 392 | 30 to 40 | 39 | 80 to 90 | 4 |
| 1 to 3 | 158 | 40 to 50 | 31 | 90 to 100 | 0 |
| 3 to 5 | 39 | 50 to 60 | 15 | Unknown | 1 |
| 5 to 10 | 49 | 60 to 70 | 16 | | |
| 10 to 20 | 31 | 70 to 80 | 5 | Total, | 815 |
| 20 to 30 | 35 | | | | |

| | | | | | |
|---|---|---|---|---|---|
| Males, | 433 | Females, | 382 | Total, | 815 |
| Single, | 702 | Married | 113 | Total, | 815 |
| White, | 811 | Colored, | 4 | Total, | 815 |

NATIVITY.

| | | | | | |
|---|---|---|---|---|---|
| Bohemia, | 4 | England, | 8 | Sweden, | 30 |
| Canada, | 10 | France, | 2 | Switzerland, | 1 |
| Chicago, Native, | 175 | Germany, | 71 | Unknown, | 4 |
| Chicago, Foreign, | 326 | Ireland, | 45 | | |
| U. S., other parts, | 109 | Norway, | 22 | Total, | 815 |
| Denmark, | 3 | Scotland, | 5 | | |

MORTALITY BY WARDS FOR THE MONTH.

| Wards. | Mortality. | Pop. in 1868. | One death in |
|---|---|---|---|
| 1 | 8 | 9,094 | 1,136½ |
| 2 | 16 | 13,074 | 817⅛ |
| 3 | 50 | 15,076 | 301½ |
| 4 | 54 | 17,796 | 329½ |
| 5 | 40 | 16,033 | 400¾ |
| 6 | 49 | 13,083 | 280 5-6 |
| 7 | 87 | 25,492 | 293 |
| 8 | 66 | 15,813 | 239 3-5 |
| 9 | 45 | 19,297 | 428 7-8 |
| 10 | 25 | 12,925 | 517 |
| 11 | 46 | 14,340 | 312 |
| 12 | 74 | 17,485 | 236¼ |
| 13 | 29 | 11,164 | 385 |
| 14 | 43 | 14,839 | 345 |
| 15 | 41 | 21,078 | 514 |
| 16 | 32 | 15,465 | 483⅓ |

| Mortality. | |
|---|---|
| Accidents, | 28 |
| Convent of Sacred Heart, | 1 |
| County Hospital, | 11 |
| Deaconess Hospital, | 1 |
| Home for Friendless, | 2 |
| Immigrants, | 44 |
| Jewish Hospital, | 1 |
| Mercy Hospital, | 1 |
| Manslaughter, | 2 |
| Protestant Orphan Asylum, | 2 |
| St. Joseph Orphan Asylum, | 13 |
| St. Luke's Hospital, | 2 |
| Suicide, | 2 |
| Total, | 815 |

---

How to Prevent Typhoid Fever.—In the British Medical Journal of March 28, Dr. Wm. Budd advocates the following means to prevent the spread of typhoid fever:

The means by which typhoid fever may be prevented from spreading are very simple, very sure, and their cost next to nothing. They are founded on the discovery that the poison by which this fever spreads is almost entirely contained in the discharges from the bowels. These discharges infect—1. The air of the sick room; 2. The bed and linen of the patient; 3. The privy and the cesspool, or the drains proceeding from them. From the privy or drain, the poison often soaks into the well, and infects the drinking-water. This last when it happens, is of all forms of fever-poisoning the most deadly. In these various ways, the infection proceeding from the bowel-discharges often spreads the fever far and wide. The one great thing to aim at, therefore, is to disinfect these discharges on their very escape from the body, and before they are carried from the sick room. This may be perfectly done by the use of disinfectants. One of the best is made of green copperas. This substance, which is used by all shoemakers, is very cheap, and may be had everywhere. A pound and a-half of green copperas to a gallon of water is the proper strength. A teacupful of this liquor, put into the night-pan every time it is used by the patient, renders the bowel discharge perfectly harmless. To disinfect the bed and body linen, and bedding generally, chloride of lime or MacDougall's powder is more convenient. These powders should be sprinkled by means of a common dredger, on soiled spots on the linen, and about the room, to purify the air. All articles of bed and body linen should be plunged, immediately on their removal, from the bed, into a bucket of water containing a tablespoonful of chloride of lime or MacDougall's powder, and should be boiled before being washed. The privy, or closet, and all drains communicating with it, should be flushed twice daily with the green copperas liquid or carbolic acid, diluted with water. In the event of death, the body should be placed, as soon as possible in a coffin sprinkled with disinfectants. Early burial is on all accounts, desirable. In towns and villages where the fever is already prevalent, the last rule should be put in force for all houses, whether there be fever in them or not, and for all public drains. As the hands of those attending on the sick often become unavoidably soiled by the discharges from the bowels, they should be frequently washed. The sick-room should be kept clean and well ventilated, day and night. The greatest possible care should be taken with regard to the drinking-water. Where there is the slightest risk of its having become tainted with fever poison, water should be got from a pure source, or should at least be boiled before being drank. Immediately after the illness is over, whether ending in death or recovery, the dresses worn by the nurse should be washed or destroyed, and the bed and room occupied by the sick should be thoroughly disinfected. These are golden rules. Where they are neglected, the fever may become a deadly scourge. Where they are strictly carried out, it seldom spreads beyond the person first attacked.

"N. B.—A yard of thin wide-width gutta percha placed under the blanket, under the breech of the patient, by effectually preventing the discharges from soaking into the bed, is a great additional safeguard."—*New Orleans Jour. of Med.*

